# Immunotherapy with nebulized pattern recognition receptor agonists restores severe immune paralysis and improves outcomes in mice with influenza-associated pulmonary aspergillosis

**DOI:** 10.1128/mbio.04061-24

**Published:** 2025-04-08

**Authors:** Jezreel Pantaleón García, Sebastian Wurster, Nathaniel D. Albert, Uddalak Bharadwaj, Keerthi Bhoda, Vikram K. Kulkarni, Mbaya Ntita, Paris Rodríguez Carstens, Madeleine Burch-Eapen, Daniela Covarrubias López, Jania Foncerrada Lizaola, Katherine E. Larsen, Lauren M. Matula, Seyed J. Moghaddam, Yongxing Wang, Dimitrios P. Kontoyiannis, Scott E. Evans

**Affiliations:** 1 Department of Pulmonary Medicine, The University of Texas M.D. Anderson Cancer Center, Houston, Texas, USA; 2 Department of Infectious Diseases, Infection Control and Employee Health, The University of Texas M.D. Anderson Cancer Center, Houston, Texas, USA; Duke University Hospital, Durham, North Carolina, USA

**Keywords:** pneumonia, influenza, aspergillosis, immunotherapy, immunomodulation, cytokines, macrophages, epithelial pathogenesis, animal models, innate immunity recognition

## Abstract

Influenza-associated pulmonary aspergillosis (IAPA) is a potentially deadly superinfection in patients with influenza pneumonia, especially those with severe disease, underlying immunosuppression, corticosteroid therapy, or requiring intensive care support. Given the high mortality of IAPA, adjunct immunomodulatory strategies remain a critical unmet need. Previously, the desensitization of pattern recognition pathways has been described as a hallmark of IAPA pathogenesis and a predictor of mortality in IAPA patients. Therefore, we studied the impact of nebulized Toll-like receptor 2/6/9 agonists Pam2 CSK4 (Pam2) and CpG oligodeoxynucleotides (ODNs) on infection outcomes and pulmonary immunopathology in a corticosteroid-immunosuppressed murine IAPA model. Mice with IAPA receiving mock therapy showed rapidly progressing disease and a paralyzed immune response to secondary *Aspergillus fumigatus* infection. Nebulized Pam2ODN was well tolerated and significantly prolonged event-free survival. Specifically, dual-dose Pam2ODN therapy before and after *A. fumigatus* infection led to 81% survival and full recovery of all survivors. Additionally, transcriptional analysis of lung tissue homogenates revealed induction of pattern recognition receptor signaling and several key effector cytokine pathways after Pam2ODN therapy. Moreover, transcriptional and flow cytometric analyses suggested increased frequencies of macrophages, natural killer cells, and T cells in the lungs of Pam2ODN-treated mice. Collectively, immunomodulatory treatment with nebulized Pam2ODN strongly improved morbidity and mortality outcomes and alleviated paralyzed antifungal immunity in an otherwise lethal IAPA model. These findings suggest that Pam2ODN might be a promising candidate for locally delivered immunomodulatory therapy to improve outcomes of virus-associated mold infections such as IAPA.

**IMPORTANCE:**

The COVID-19 pandemic has highlighted the significant healthcare burden, morbidity, and mortality caused by secondary fungal pneumonias. Given the heightened prevalence of severe viral pneumonias, such as influenza, and poor outcomes of secondary mold pneumonias, adjunct immunotherapies are needed to prevent and treat secondary infections. We herein demonstrate severely paralyzed immunity to secondary *Aspergillus fumigatus* infection in a corticosteroid-immunosuppressed mouse model of influenza-associated pulmonary aspergillosis (IAPA), partially due to dysregulated pathogen-sensing pathways. To overcome immune paralysis and IAPA progression, we used a dyad of nebulized immunomodulators (Toll-like receptor agonists). Nebulized immunotherapy significantly improved morbidity and mortality compared to mock therapy, increased frequencies of mature mononuclear phagocytes and natural killer cells in the lung, and stimulated antimicrobial signaling. Collectively, this proof-of-concept study demonstrates the feasibility and efficacy of locally delivered immunomodulatory therapy to alleviate virus-induced immune dysregulation in the lung and improve outcomes of post-viral mold pneumonias such as IAPA.

## INTRODUCTION

Lower respiratory tract infections (LRTIs) with respiratory viruses, such as influenza A virus (IAV), are associated with significant morbidity and mortality, especially in immunocompromised hosts (1). Compounding the poor outcomes of viral LRTIs, secondary pneumonias caused by bacteria and, as increasingly appreciated, by fungi, constitute a significant source of morbidity and mortality after viral LRTIs (2, 3). Invasive pulmonary aspergillosis, predominantly caused by *Aspergillus fumigatus* (AF), is the most common fungal superinfection after viral pneumonia (3). Concerningly, about 20% of critically ill influenza patients develop influenza-associated pulmonary aspergillosis (IAPA), and the incidence is even higher in patients with additional underlying risk factors for invasive aspergillosis and those receiving glucocorticosteroids for the management of respiratory failure (4, 5). Even with modern intensive care and potent antifungal agents, mortality rates of IAPA remain as high as 50%, underscoring an unmet need for novel adjunct therapeutic approaches, including immunotherapy (5, 6).

The immunopathogenesis of virus-associated pulmonary aspergilloses is thought to be driven by a coalescence of virus-induced damage to the epithelial barrier, pulmonary hyperinflammation, and several hallmarks of local and systemic immune dysregulation and impairment (7–11). Common surrogates of pulmonary immune paralysis in IAPA patients and murine IAPA models include impaired recruitment and maturation of innate effector cells, unfavorable polarization and exhaustion of adaptive cellular immunity, and attenuated pattern recognition receptor (PRR) signaling (12–15). For instance, IAV infection reduced transcription levels of several PRRs in a murine IAPA model, including Toll-like receptors (TLRs) and C-type lectin receptors involved in fungal recognition (13). Likewise, transcriptional immune profiling of bronchoalveolar lavage (BAL) samples from IAPA patients revealed the downregulation of several genes encoding proteins involved in fungal recognition and killing, including TLR2, a key receptor for the recognition of AF conidia (14). Moreover, a recent study in IAPA patients admitted to the intensive care unit identified the downregulation of PRR genes as a significant predictor of increased mortality (15), further corroborating the significance of impaired PRR signaling in the pathogenesis of IAPA. Therefore, immunomodulatory strategies restoring and enhancing pulmonary PRR signaling might be a promising adjunct approach to improve IAPA outcomes.

Prior studies by our group revealed significantly improved morbidity and mortality, enhanced epithelial resistance and viral clearance, and attenuated viral immune injury after inhaled immunomodulatory therapy with the synergistic TLR2/TLR6 and TLR9 agonists Pam2CSK4 and CpG oligodeoxynucleotides M362 (Pam2ODN) in murine models of various pneumonias, including IAV, paramyxovirus, and coronavirus infections (16–19). Furthermore, Pam2ODN provided a potent therapeutic benefit and facilitated rapid fungal killing in mice with underlying chemotherapy-induced immunosuppression and invasive pulmonary aspergillosis (20). Given these encouraging results in murine mono-infection models and the critical role of PRR signaling in the pathogenesis of IAPA, we herein studied Pam2ODN as an adjunct immunotherapy in mice with IAPA. We found that immunomodulatory treatment with nebulized Pam2ODN strongly improved infection outcomes and enhanced antimicrobial defense in our otherwise lethal and severely immuno-paralyzed corticosteroid-immunosuppressed IAPA model.

## RESULTS

### CA-immunosuppressed mice IAPA display a state of severe immune paralysis

**Fig 1:**
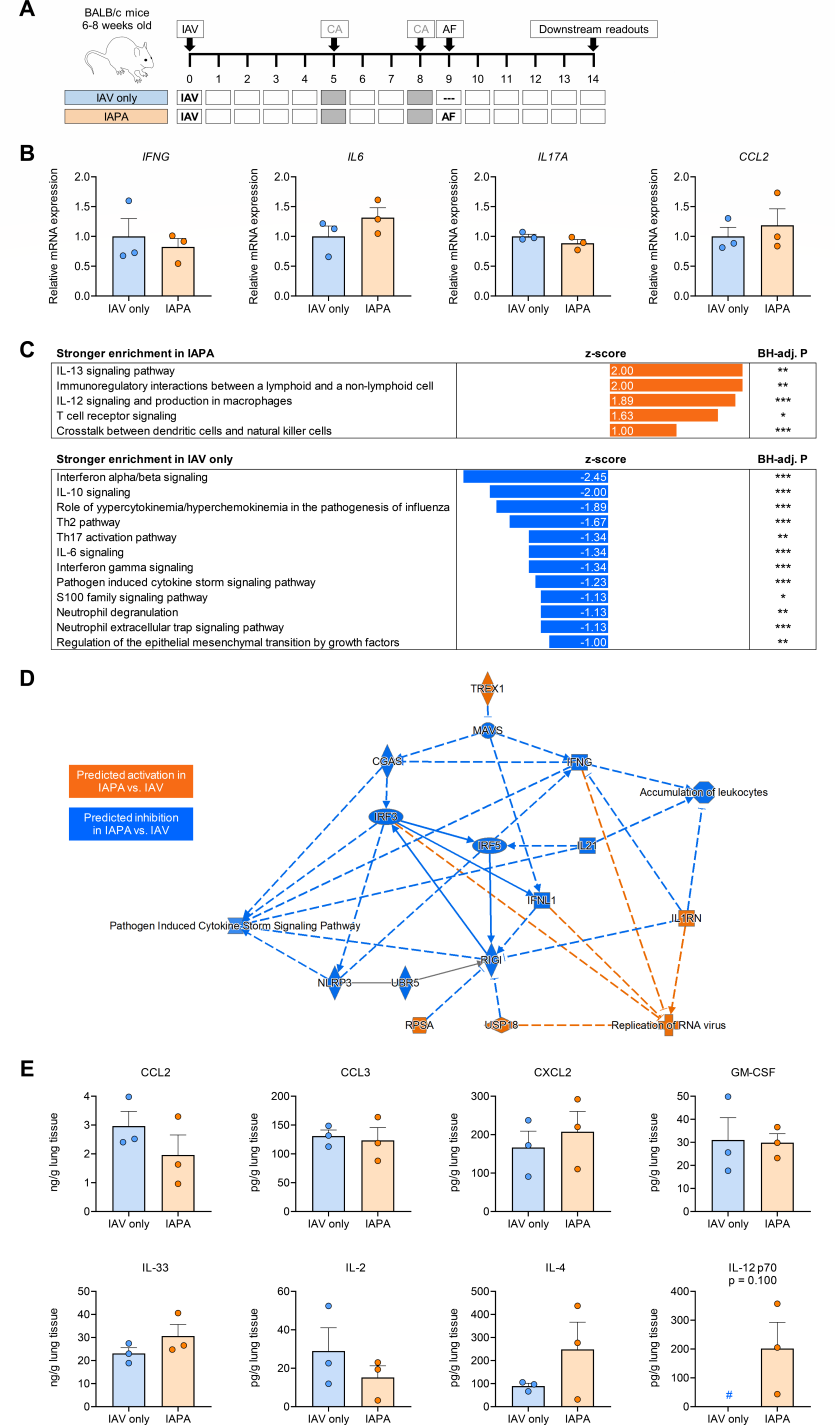
Mice with IAPA display an immuno-paralytic phenotype with dysregulated pattern recognition receptor signaling and minimal incremental inflammatory response to AF superinfection. (A) Timeline of experimental interventions. (B) Expression of cytokine genes with known roles as master regulators of antifungal immunity in lung tissue homogenates of mice with IAPA compared to those with IAV mono-infection. Unpaired *t*-test. (C) Comparison of canonical pathway enrichment based on transcriptional responses (nCounter Host Response panel) in the lung tissue of mice with IAPA compared to those with IAV mono-infection. Differential pathway enrichment was defined as an absolute *z*-score ≥ 1 and a Benjamini-Hochberg (BH)-adjusted *P*-value ≤ 0.05. (D) Network of transcriptional changes to the pulmonary immune environment in mice with IAPA versus those with IAV mono-infection as predicted by Ingenuity Pathway Analysis. (E) Comparison of cytokine and chemokine concentrations in mice with IAPA versus those with IAV mono-infection. Mann-Whitney *U* test. # indicates that all three replicates in the “IAV-only” group were below the lower limit of detection (~30 pg/g lung tissue). TNF-α, IFN-γ, IL-6, IL-17A, and CCL4 were below the limit of detection in most or all mice, regardless of AF superinfection (not shown). (B–E) *N* = 3 mice per group and readout. Columns and error bars represent means and standard errors of the means, respectively. CA, cortisone acetate; C(X)CL, C-(X-)C motif chemokine ligand; GM-CSF, granulocyte macrophage colony stimulating factor; IFN, interferon; IL, interleukin; and TNF, tumor necrosis factor.

IAV-infected and CA-immunosuppressed mice showed rapidly progressing disease upon AF superinfection, i.e., induction of IAPA (Fig. 1A) (21). Despite severe infection, reverse transcription-quantitative polymerase chain reaction (RT-qPCR) analysis of lung tissue from mice with IAPA showed minimal induction of genes associated with key antifungal effector responses, such as type-1 T-helper cell (Th1) (*IFNG*) and Th17 (*IL17A*) responses, mononuclear inflammation (*IL6*), and recruitment of innate effector cells (e.g., *CCL2*), with relative expression levels of 0.82–1.32 in IAPA versus IAV-only groups (Fig. 1B).

nCounter-based transcriptional analysis of lung tissue further corroborated a state of severe immune paralysis in mice with IAPA, with minimal inflammatory response to AF superinfection compared to IAV infection only. Specifically, a comparison of absolute normalized transcript counts in mice with IAV infection only versus those with IAPA revealed minimal differences between the two groups. Specifically, key markers of antifungal immunity (cytokines, chemokines, maturation markers, and cytotoxic effectors) showed low expression levels when compared to epithelial markers and general stress response markers (e.g., hsp signaling), regardless of co-infection status (Fig. S1).

Pathway enrichment analysis confirmed only modest induction (*z*-score, 1–2) of a few pathways associated with intercellular signaling and adaptive immune activation upon AF superinfection (Fig. 1C). These signals were counterbalanced by significant suppression of several immune pathways in mice with IAPA versus those with IAV infection only, especially key effector cytokine pathways (e.g., IL-6, IFN-γ, and type-1 interferons) and pathways associated with neutrophilic effector responses (Fig. 1C). Moreover, AF superinfection was associated with the suppression of several mediators of PRR signaling pathways, including pathways associated with viral control (e.g., RIG-I, CGAS, IRF3, and IRF5) (Fig. 1D). These findings suggest a state of severe immune paralysis and impaired pathogen control in mice with IAPA.

Consistent with this observation, phenotypic validation of cytokine concentrations in lung tissue homogenates revealed largely comparable concentrations of innate effector cytokines in mice with IAPA versus those with IAV infection only. Specifically, no increased production of key chemokines and growth factors associated with recruitment of innate effector cells (CCL2, CCL3, CXCL2, GM-CSF, and IL-33) was seen, with mean inter-group ratios of 0.66–1.33 (*P* = 0.400–1.000) (Fig. 1D). Moreover, type-1 and type-17 T-helper cell signature cytokines IFN-γ and IL-17A were below the detectable range in most animals, regardless of AF superinfection (data not shown), and pulmonary IL-2 concentrations tended to be lower in mice with IAPA compared to those with IAV infection only (Fig. 1D). Consistent with our nCounter-based pathway enrichment analysis, IL-12 p70 was the only tested cytokine that was markedly elevated after AF superinfection (Fig. 1D).

Independently, we assayed the cellular composition of the lung using the NanoString cell type profiling module. Estimating the abundance of nine distinct leukocytic populations, we found marginal percentual changes between the IAPA- and IAV-only groups in the proportion of all cell types (Fig. S2A and B). Collectively, these findings corroborate a state of severe immune paralysis, with minimal incremental inflammation and no significant changes in cellular composition after AF superinfection despite the severe “clinical” manifestation of IAPA in our model.

### Immunotherapy with nebulized Pam2ODN improves clinical outcomes in immunosuppressed mice with IAPA

**Fig 2:**
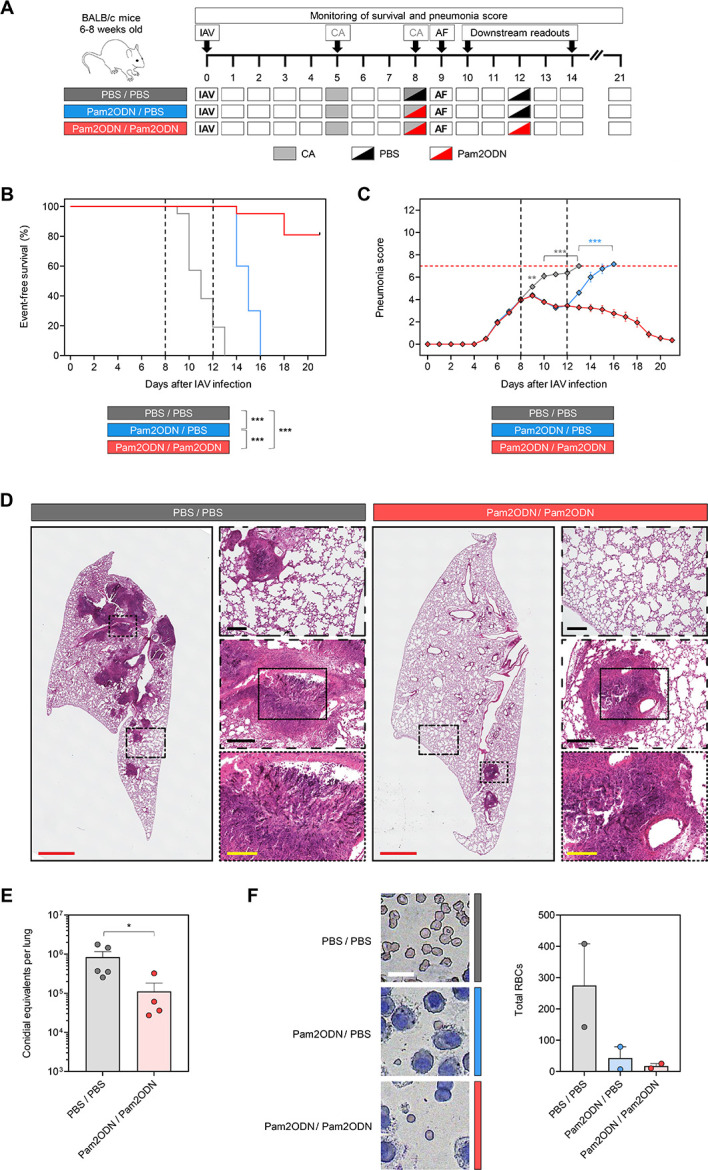
Dual-dose immunotherapy with nebulized Pam2ODN strongly improves clinical outcomes in CA-immunosuppressed mice with IAPA. (A) Outline of experimental interventions. (B) Kaplan-Meier curves comparing event-free survival in CA-immunosuppressed mice with IAPA according to the treatment arm. *N* = 20–21 mice per group assessed across three independent experiments. Mantel-Cox log-rank test. (C) Pneumonia scores over time according to the treatment arm. Mice that have reached the morbidity/mortality endpoint prior to the time of assessment are excluded. Multi-group comparisons at each time point were performed using one-way analysis of variance. (D) Representative H&E-stained lung tissue sections on day 14 (5 days after AF infection). Scales: red, 2 mm; black, 200 µm; and yellow, 100 µm. (E) Fungal burden assessed by quantitative PCR on day 14. *N* = 4–5 mice per treatment arm. Mann-Whitney *U* test. (F) Representative micrographs of RBC burden in bronchoalveolar lavage and quantitative analysis of total RBCs per field according to the treatment arm (day 14). *N* = 2 mice per treatment arm. Scale: 15 µm. (C, E, and F) Means and standard errors of the mean (error bars) are shown. CA, cortisone acetate; H&E, hematoxylin and eosin; PBS, phosphate-buffered saline; Pam2ODN, Pam-2 CSK4 + CpG oligodeoxynucleotides M362; PCR, polymerase chain reaction; and RBC(s), red blood cell(s).

To overcome immune paralysis in mice with IAPA and re-invigorate PRR signaling, mice received nebulized immunostimulatory Pam2ODN treatment either before AF superinfection or both before and after superinfection (Fig. 2A). All mock-treated corticosteroid-immunosuppressed mice with IAPA reached the combined morbidity/mortality endpoint by day 13, i.e., within 4 days of AF infection. Single-dose Pam2ODN therapy on day 8 led to universal event-free survival until day 13, but all mice reached the morbidity/mortality endpoint by day 16 (Fig. 2B). In contrast, dual-dose Pam2ODN therapy on days 8 and 12, i.e., both before and after *A. fumigatus* infection, led to 80% event-free survival until day 21 (Fig. 2B, *P* < 0.001 versus all other groups). Notably, all survivors fully recovered by day 21 (mean pneumonia score = 0.4; Fig. 2C).

Consistent with these clinical trends, hematoxylin and eosin-stained whole-lung sections revealed a significantly reduced number and extent of fungal infiltrates (Fig. 2D) and a 7.5-fold reduction in fungal burden (Fig. 2E) in Pam2ODN-treated mice compared to the PBS-treated group. Moreover, microscopic analysis of BAL fluid corroborated markedly reduced erythrocyte counts as a surrogate of hemorrhagic lesions in Pam2ODN-treated animals with IAPA (means, 43 and 18 after single-dose and dual-dose Pam2ODN, respectively) compared to mock-treated infected animals (mean, 275; Fig. 2F). Expectedly, diffuse alveolar damage due to IAV infection was not attenuated by Pam2ODN immunotherapy that was initiated 8 days after viral infection (Fig. 2D).

### Immunotherapy with Pam2ODN re-invigorates PRR and downstream cytokine signaling

**Fig 3:**
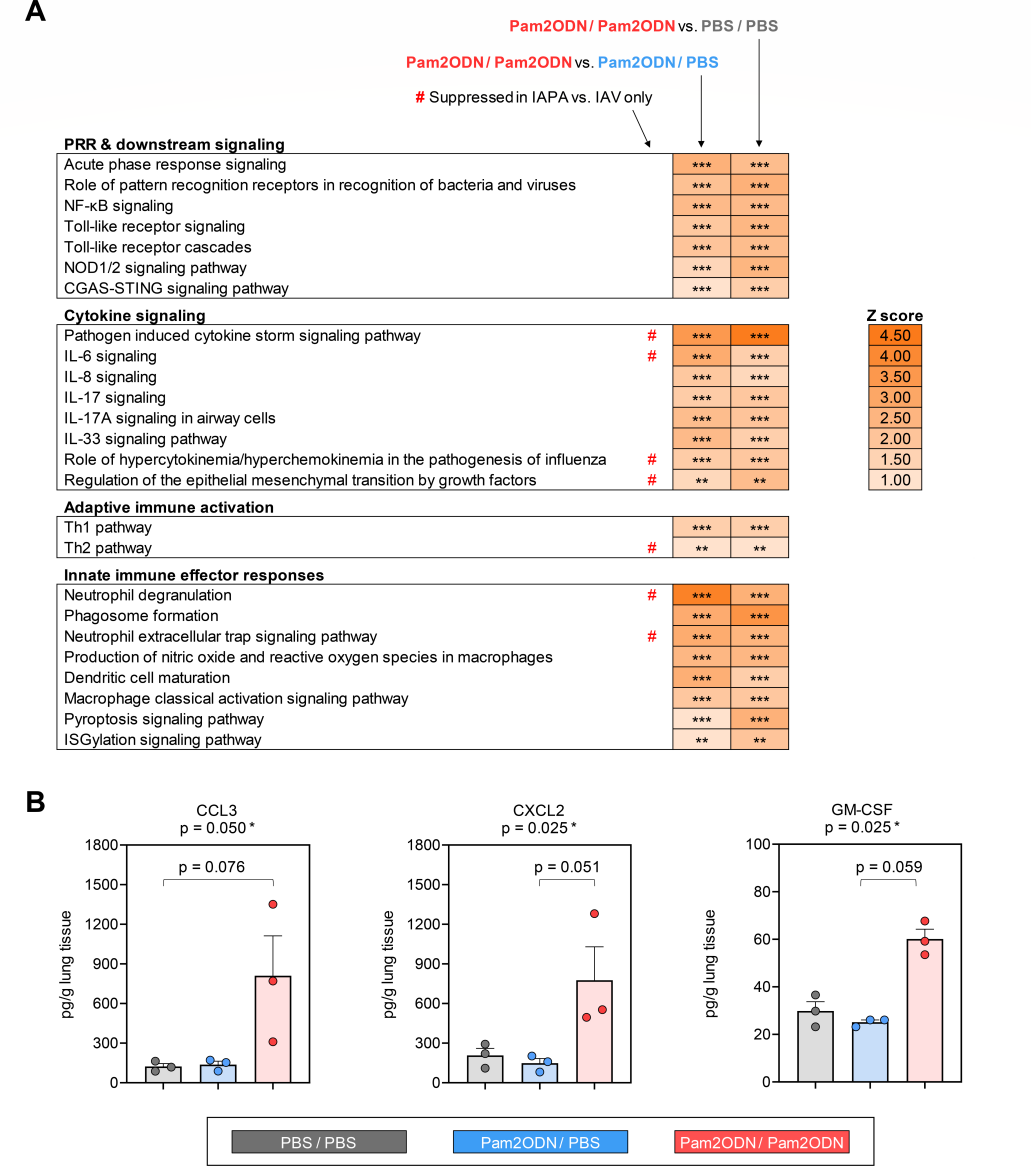
Dual-dose immunotherapy with nebulized Pam2ODN before and after AF superinfection enhances innate immune defense in CA-immunosuppressed mice with IAPA. (A) Comparison of canonical pathway enrichment based on transcriptional responses (nCounter Host Response panel) in lung tissue homogenates of mice with IAPA according to the treatment arm. (B) Concentrations of selected cytokines/chemokines in lung tissue homogenates of mice with IAPA according to the treatment arm. *P*-values for multi-group comparisons (Kruskal-Wallis test) and pairwise comparisons approaching significance (Dunn’s post-test) are shown above and within the panels, respectively. All other tested cytokines were either below the limit of detection in most mice regardless of the treatment arm (CCL4, IFN-γ, IL-6, IL-12 p70, IL-17A, and TNF-α) or not significantly different between the treatment arms (CCL2, IL-2, IL-4, and IL-33) (Table S1). (A and B) All analyses were performed on day 14 (5 days after AF infection). *N* = 3 mice per group and readout. CGAS-STING pathway, cyclic GMP-AMP synthase/stimulator of interferon genes pathway; C(X)CL, C-(X-)C motif chemokine ligand; GM-CSF, granulocyte macrophage colony stimulating factor; IFN, interferon; IL, interleukin; NF-κB, nuclear factor kappa-light-chain-enhancer of activated B-cells; NOD1/2, nucleotide-binding oligomerization domain-containing protein 1/2; PBS, phosphate-buffered saline; Pam2ODN, Pam-2 CSK4 + CpG oligodeoxynucleotides M362.

To comprehensively characterize the pulmonary immune environment in mock- and Pam2ODN-treated mice with IAPA, we performed nCounter and pathway enrichment analysis. Dual-dose Pam2ODN therapy led to significant induction of PRR (TLR, NOD1/2, and cGAS/STING) and NF-κB signaling pathways, along with the induction of key innate effector cytokine pathways (e.g., IL-6, IL-8, IL-17A, and IL-33) (Fig. 3A). These changes were associated with several signals of enhanced innate immune cell effector responses, including enhanced DC maturation, macrophage activation, phagosome formation, and oxidative burst, as well as enhanced neutrophil degranulation and neutrophil extracellular trap signaling (Fig. 3A). Furthermore, pathway enrichment analysis suggested modest enhancement of T-helper signaling (Fig. 3A). Notably, 7 out of the 12 pathways significantly suppressed in mice with IAPA compared to those with IAV mono-infection (Fig. 1B) were restored by dual-dose Pam2ODN therapy (Fig. 3A).

An integrative predicted network of gene- and pathway-level expression changes corroborated enhanced PRR and downstream signaling (e.g., MYD88 and RelA) after dual-dose Pam2ODN treatment. Furthermore, Ingenuity Pathway Analysis revealed the induction of several key effector cytokines (e.g., TNF-α, IFN-γ, IL-1β, and IL-6) and chemokines (CXCL2 and CXCL3) that are associated with antifungal immune enhancement, mobilization of innate effector cells, and increased pathogen clearance (Fig. S3) (22).

Of note, recent work (23) underscored that some cytokine pathways might time-dependently elicit either protective or detrimental roles in the immune defense against IAPA. Specifically, exuberant early interferon signaling was identified as a major driver of immunopathology in IAPA. Therefore, we performed RT-qPCR studies on lung tissue from mice with IAPA on day 10, i.e., 2 days after the first Pam2ODN dose. Here, we found that Pam2ODN therapy suppressed the early expression of interferon gamma (fold change 0.29, *P* = 0.016) compared to mock-treated mice with IAPA. Moreover, there was no indication of early hyper-inflammation after the first Pam2ODN dose, as evidenced by the unaltered expression of TNF-α and CCL2, IL-6, and IL-17 (Fig. S4 and data not shown). Consistent with previous studies (24), Pam2ODN did increase early (day 10) expression of genes associated with epithelial pathogen clearance (e.g., SAA3, *P* = 0.034).

Collectively, our transcriptional analyses suggested time-dependent effects of Pam2ODN in mice with IAPA, with early attenuation of interferon gamma-driven immunotoxicity and enhancement of epithelial resistance preceding later induction of pathogen sensing and innate effector responses after the administration of the second Pam2ODN dose.

### Pam2ODN promotes the accumulation of mature mononuclear phagocytes, natural killer cells, and T cells in the lung

Next, we tested the induction of antifungal effector cytokines on protein level by Luminex-based analysis of lung tissue homogenates. While less sensitive than our transcriptional analyses, we found strong induction of cytokines associated with the mobilization of innate effector cells, especially mononuclear phagocytes, in mice with IAPA that received dual-dose Pam2ODN therapy compared to those receiving the mock treatment or single-dose Pam2ODN (Table S1). Specifically, strong and significant elevations of CCL3 (6.57-fold compared to mock therapy, *P* = 0.025), CXCL2 (3.74-fold, *P* = 0.050), and GM-CSF (2.01-fold, *P* = 0.025) were found after dual-dose Pam2ODN therapy (Fig. 3B). Consistent with this observation, Ingenuity Pathway Analysis of transcriptional data predicted increased mobilization of mononuclear phagocytes, natural killer cells, and (T) lymphocytes after dual-dose Pam2ODN compared to mock therapy and single-dose Pam2ODN treatment (Fig. S3).

**Fig 4:**
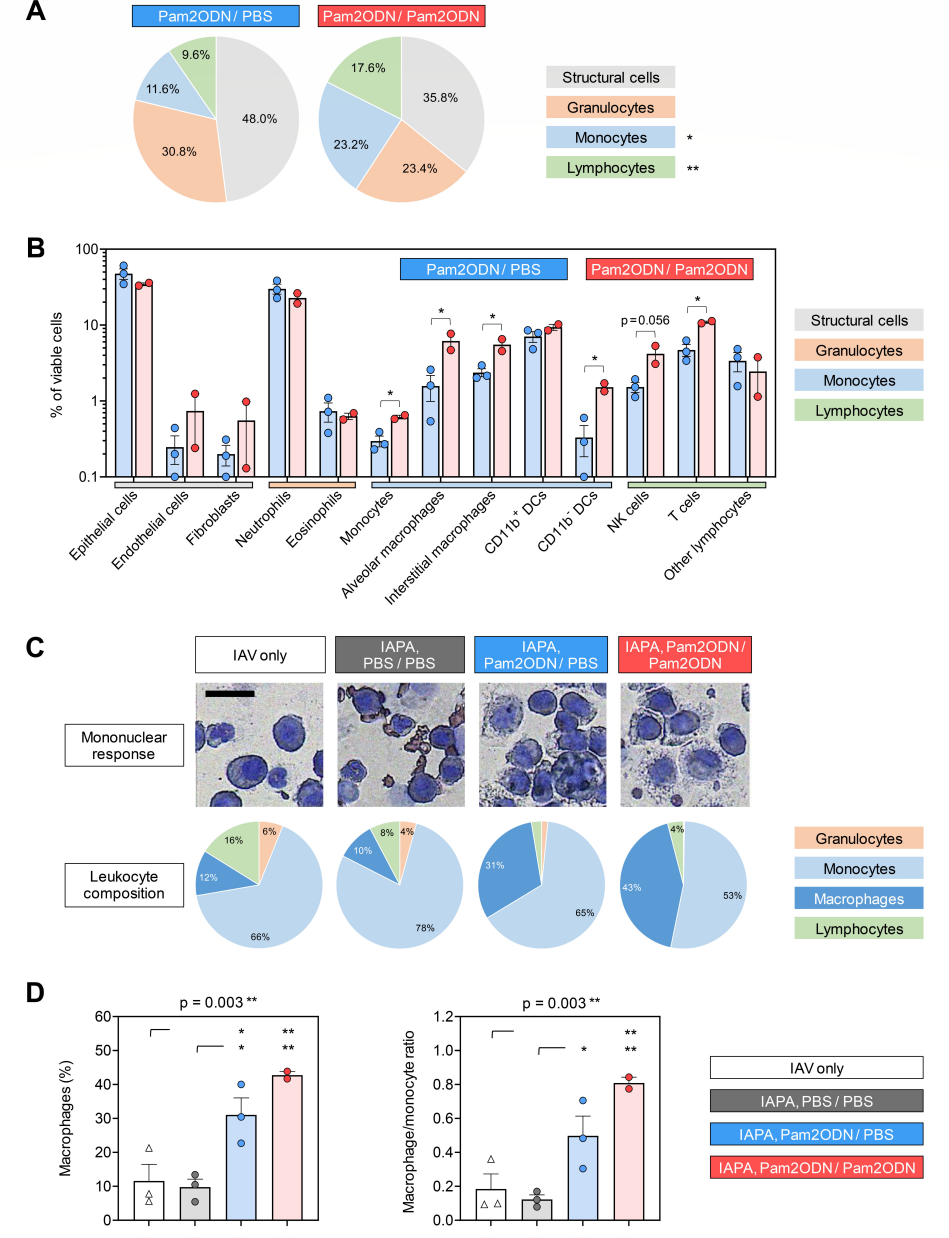
Immunotherapy with nebulized Pam2ODN promotes pulmonary recruitment of mature mononuclear effector cells in mice with IAPA. (A and B) Flow cytometric analysis of immune cells and structural cell populations (epithelium, endothelium, and fibroblasts) in lung tissue homogenates of mice with IAPA receiving single-dose versus dual-dose Pam2ODN therapy. Unpaired *t*-test. All significant results were confirmed to have false discovery rates < 0.2 (Benjamini-Hochberg method). (C) Representative images and quantitative analysis of leukocyte subsets in BAL according to the infection and treatment arm. Subsets ≤ 3% are not labeled. Scale: 20 µm. (D) Proportions of macrophages among leukocytes and macrophage/monocyte ratios in BAL according to the infection and treatment arm. One-way analysis of variance with Tukey’s post-test. (A–D) *N* = 2–3 mice per treatment arm and assay. All analyses were performed on day 14 (5 days after AF infection). CD, cluster of differentiation; DCs, dendritic cells; NK cells, natural killer cells; PBS, phosphate-buffered saline; and Pam2ODN, Pam-2 CSK4 + CpG oligodeoxynucleotides M362.

Given the strong impact of the second (post-AF) Pam2ODN dose on pulmonary secretion of cytokines and chemokines associated with the recruitment of innate effector cells, we used flow cytometry to compare the cellular composition of lung tissue from mice that received dual-dose versus single-dose Pam2ODN therapy. Compared to single-dose therapy, dual-dose Pam2ODN led to globally increased leukocyte content in lung tissue homogenates (64.2% versus 52% of total viable cells, excluding red blood cells and platelets; Fig. 4A). Particularly, mice with IAPA that received dual-dose Pam2ODN therapy showed markedly increased pulmonary frequencies of alveolar macrophages (6.2% versus 1.6% of total viable cells, *P* = 0.042), interstitial macrophages (5.3% versus 2.4%, *P* = 0.031), CD11b^-^ dendritic cells (1.5% versus 0.3%, *P* = 0.018), natural killer cells (4.2% versus 1.5%, *P* = 0.056), and T lymphocytes (10.9% versus 4.7%, *P* = 0.011) compared to mice that received single-dose Pam2ODN (Fig. 4B). Of note, due to the low number of non-moribund animals, flow cytometric data from the mock-treated group (PBS/PBS) could not be obtained.

To further validate Pam2ODN-induced changes to the pulmonary leukocyte repertoire, we performed microscopic analysis of leukocyte composition in bronchoalveolar lavage fluid (BALF). Here, we found a significant increase in macrophages from 10% in mock-treated mice with IAPA to 31% and 43% in those receiving single- and dual-dose Pam2ODN therapy, respectively (*P* = 0.003; Fig. 4C and D). This was paralleled by an increase in the macrophage/monocyte ratio from 0.13 (mock treatment) to 0.48 (single-dose Pam2ODN) and 0.81 (dual-dose Pam2ODN), respectively (*P* = 0.003; Fig. 4E). These findings corroborate that immune protection by Pam2ODN therapy is, at least in part, driven by increased mobilization and maturation of mononuclear phagocytes.

**Fig 5:**
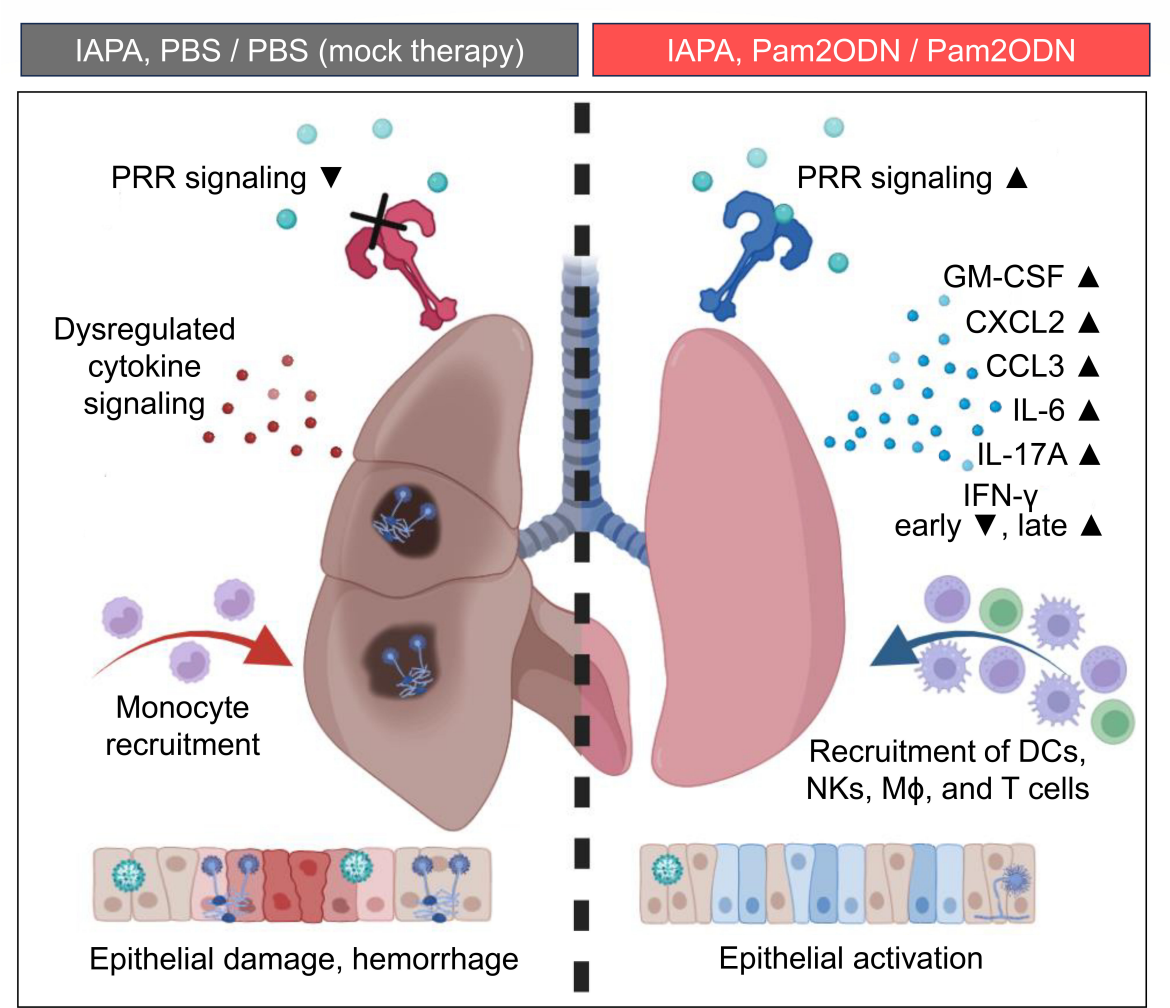
Immunotherapy with nebulized Pam2ODN alleviates pulmonary immunopathology to improve outcomes in immunosuppressed mice with IAPA. Schematic summarizing immuno-protective effects of nebulized Pam2ODN therapy in mice with IAPA. C(X)CL, C-(X-)C motif chemokine ligand; GM-CSF, granulocyte-macrophage colony-stimulating factor; IFN, interferon; IL, interleukin; Mϕ, macrophages; NK, natural killer cells; PBS, phosphate-buffered saline; Pam2ODN, Pam-2 CSK4 + CpG oligodeoxynucleotides M362.

Altogether, our data support a model whereby immunotherapy with nebulized Pam2ODN attenuates immunotoxicity, re-invigorates PRR signaling, stimulates mobilization of key immune cell populations, induces innate effector cytokine responses, and enhances epithelial resilience (Fig. 5). Collectively, these changes to the pulmonary immune environment alleviate infection-induced immune paralysis and restore anti-AF defense in mice with IAPA, thereby attenuating fungal invasion and improving morbidity/mortality outcomes.

## DISCUSSION

The COVID-19 pandemic has highlighted the significant healthcare burden, morbidity, and mortality caused by secondary fungal pneumonias. Given the heightened prevalence of respiratory viruses associated with severe secondary mold pneumonias, novel adjunct strategies are needed to improve the outcomes of these infections. Here, we studied locally delivered (nebulized) PRR agonists as an immunotherapeutic intervention to improve the outcome of IAPA in glucocorticosteroid-immunosuppressed mice by attenuating epithelial pathogenesis and pulmonary immune paralysis.

In the first part of this study, we characterized the natural response to AF superinfection in mice with underlying influenza and high-dose corticosteroid therapy. Our observation of a paralyzed or even suppressed immune environment in mice with IAPA versus those with influenza only aligns with extensive evidence from immune monitoring studies in human patients with viral pneumonia and post-viral mold infections. For instance, we found a notable overlap of the most strongly suppressed transcriptional pathways (e.g., type-1 interferon signaling, IFN-γ signaling, IL-6 signaling, and various pathways related to neutrophil effector responses) between our model and a prior study in human patients with IAPA versus influenza only using a similar methodology (nCounter-based transcriptomics with pathway enrichment analysis) (14). Our finding of a largely anergic immune state and impaired PRR sensing is also consistent with a more recent publication by the same group showing minimal inflammatory responses to bacterial co-infection in influenza patients with and without IAPA (23). Additionally, our finding of impaired viral pathogen sensing via RIG-I, cGAS, and their downstream effectors in mice with IAPA versus those with influenza mono-infection aligns well with the mutual impairment of viral and fungal PRR pathways previously described in an *in vitro* model of AF and cytomegalovirus co-infection (24).

Our model used CA immunosuppression to recapitulate a common risk factor in human IAPA patients and facilitate robust infection with a relatively low AF inoculum. While it is conceivable that CA contributed to the lack of a pronounced inflammatory response to AF superinfection in our model, there are several lines of evidence suggesting that CA was not a major confounder of our findings. On the one hand, potent innate immune cell recruitment and cytokine release had been reported previously in single-infection pulmonary aspergillosis models using similar high-dose glucocorticosteroid regimens (25, 26). On the other hand, several clinical immune phenotyping studies suggested that hallmarks of paralyzed antifungal immunity in patients with viral pneumonia were largely independent of corticosteroid therapy (14, 27). Furthermore, our findings of both attenuated PRR signaling and transcriptional surrogates of impaired neutrophil effector responses in mice with IAPA align with data from Liu and colleagues (13), who used an IAPA model without pharmacological immunosuppression. Similarly, Lee and colleagues (28) found that inflammation in a non-immunosuppressed IAPA model was mainly driven by the underlying IAV infection, whereas secondary aspergillosis had a limited contribution to inflammatory responses.

To overcome dysfunctional PRR signaling and immune paralysis in our CA-immunosuppressed IAPA model, we tested immunomodulatory therapy with nebulized Pam2ODN, which allowed most mice to clear IAPA infection and fully recover from infection-induced distress after only two Pam2ODN doses and without concomitant antiviral or antifungal therapy. Mechanistically, dual-dose Pam2ODN immunotherapy attenuated early interferon gamma-driven immunotoxicity, enhanced PRR and downstream signaling, induced essential antifungal effector cytokine pathways, and promoted recruitment, differentiation, and maturation of mononuclear phagocytes. Thereby, Pam2ODN immunotherapy targets multiple common immune deficits in sequential inter-kingdom infections (7–9).

Interestingly, single-dose Pam2ODN before AF superinfection elicited minimal changes to pro-inflammatory cytokine release. This might be due to the timing of the first Pam2ODN dose after the peak of IAV-induced distress and before AF superinfection. Additionally, given that PRR agonists are known inducers of trained immunity (29), the leukocyte-driven component of Pam2ODN-induced immune augmentation likely benefits from repeat exposure to the agent, as seen previously in mice treated with individual nebulized exposures to Pam2, ODN, and other PRR agonists at increasing concentrations (16).

While the present study focused on leukocyte-driven pulmonary immune augmentation, early transcriptional induction of the assayed epithelial defense genes is consistent with extensive prior evidence of Pam2ODN as an inducer of epithelial resistance in various preclinical studies of bacterial and viral mono-infections. This observation is partially due to the capacity of Pam2ODN to stimulate mitochondrial production of reactive oxygen species (ROS) and induce ROS-dependent epithelial STAT3 signaling (30, 31).

Consistent with the favorable tolerability data for nebulized Pam2ODN (PUL-042) in human patients with viral pneumonia (ClinicalTrials.gov NCT04312997) and our extensive prior preclinical work in various single-infection pneumonia models, immunotherapy was well tolerated in our IAPA model, and no evident immunotoxicities were seen. In fact, the conceivable tolerability advantages of topically delivered immunomodulators compared to systemic immune enhancers studied as antifungal immunotherapies might be particularly relevant in a background of underlying viral infections that are often associated with systemic hyperinflammation. Therefore, our favorable tolerability and efficacy data might encourage further comparative investigations of inhaled versus systemic administration of immunomodulators in primary or secondary mold pneumonias or polymicrobial respiratory tract infections. For instance, immune checkpoint inhibitors or recombinant GM-CSF showed promise as antifungal immunotherapies (32–35) and are available as (investigational) nebulized immune agents (36, 37).

Furthermore, the benefits of Pam2ODN might also apply to other co- and sequential infections. Feys and colleagues (14) found that several signals of immune paralysis, including suppressed TLR2 expression, were shared between patients with IAPA- and COVID-19-associated pulmonary aspergillosis. Similarly, as discussed above, strong mutual impairment of PRR signaling and anti-infective effector responses was found in an *in vitro* model of *A. fumigatus* and cytomegalovirus co-infection (38). Additionally, post-viral impairment of antimicrobial signaling has also been described in response to other fungal stimuli. For instance, weak PRR expression and dysfunctional phagocytosis after IAV infection were encountered in mice challenged with either *A. fumigatus* conidia or yeast zymosan (13). Likewise, patients with moderate COVID-19 showed surrogates of impaired TLR signaling in response to *Rhizopus arrhizus*, the most common cause of COVID-19-associated mucormycosis (11, 27). Collectively, these studies suggest that dysfunctional PRR signaling may be a hallmark of immune dysregulation that is broadly applicable to many viral and fungal inter-kingdom infection settings, inviting further studies of Pam2ODN immunotherapy in other co- and sequential infection models, e.g., highly lethal mold pneumonias after LRTIs due to respiratory syncytial virus (39). Moreover, patients with IAPA and bacterial co-infection were shown to have minimal incremental pro-inflammatory pulmonary cytokine responses to bacterial co-pathogens, corroborating a severely paralyzed immune state (40). As Pam2ODN confers robust protection against virulent gram-positive and gram-negative bacteria (16), nebulized Pam2ODN might be a particularly attractive immunotherapeutic approach in patients with complex polymicrobial pneumonias due to its potential triple activity against viral, fungal, and bacterial co-pathogens.

This proof-of-concept study has several limitations. Immune impairment and immunomodulatory therapy were studied without the complexities of conventional antiviral or antifungal agents that would modulate infection severity and inflammation but might also elicit both immunostimulatory and inhibitory effects (21, 41–43). Furthermore, our study utilized a single IAV and AF strain, and therefore, it does not reflect the considerable strain-to-strain variability. Despite the consistency of the underlying immune paralysis phenotype in our IAPA model with both previously published mouse models using different IAV and AF strains and real-life human patient data, confirmatory evidence for the benefits of Pam2ODN therapy against sequential infection with additional strains would be warranted. Moreover, the high mortality and rapid disease progression in the mock-treated IAPA group greatly limited sample availability for our various downstream readouts, especially on day 14 (day 5 after AF superinfection), reducing statistical power and not allowing us to perform all assays on the mock-treated cohort. Finally, secondary mold pneumonias after respiratory viral infections most commonly affect patients with underlying classical risk factors for invasive fungal infections, such as uncontrolled diabetes mellitus, hematological malignancies, transplant history, or ongoing immunosuppressive therapies. Besides corticosteroid therapy, such comorbidities were not recapitulated in our study using *a priori* healthy inbred mice.

Collectively, our findings corroborate that IAPA is associated with a severely immuno-paralytical state, aligning with prior studies in IAPA mouse models and human patients with post-viral mold pneumonia. Immunotherapy with nebulized Pam2ODN was well tolerated and strongly improved clinical outcomes in our otherwise highly lethal IAPA model, reduced fungal burden, enhanced mature mononuclear phagocyte responses, and induced antimicrobial signaling. These findings suggest a promising therapeutic potential of nebulized immunomodulators to mitigate pulmonary immune paralysis and improve IAPA outcomes.

## MATERIALS AND METHODS

### Murine infection model

Female BALB/c mice, aged 8–10 weeks, were infected with a sublethal dose (<LD_10_; ~25,000 plaque-forming units) of a mouse-adapted influenza A/Hong Kong/1968 (H3N2) strain, delivered by aerosolization, as previously described (17, 21). On days 5 and 8 after influenza infection, mice received two intraperitoneal injections of 10 mg cortisone acetate (CA; Sigma-Aldrich). On day 9, mice were challenged intranasally with 50,000 AF-293 conidia or sterile saline (mock infection). For therapeutic experiments, mice received a 30-minute nebulization of either phosphate-buffered saline (PBS; mock treatment) or Pam2ODN (4 µM Pam2CSK4 + 1 µM ODN M362) before (single dose, day 8) or before and after AF infection (dual dose, days 8 and 12). Infection severity was scored daily using a clinical pneumonia score (0 = healthy to 12 = moribund), as described previously (44). To assess therapeutic efficacy, a combined morbidity and mortality endpoint was used, with an event defined as either death or reaching a pneumonia score ≥7, a score indicating considerable distress and a high likelihood of death within 72 hours in the absence of anti-infective therapy (21, 44). This approach allowed us to obtain immunologic samples from non-moribund mice without compromising the primary endpoint analysis.

### Histopathological analyses

Representative mouse lung tissue sections were fixed and stained with hematoxylin and eosin as previously described (21). Briefly, mouse lungs were fixed intratracheally and incubated for 1 day with 10% formalin, transferred to 70% ethanol, and embedded in paraffin. Formalin-fixed, paraffin-embedded tissue blocks were then cut into 10 µm sections, mounted, deparaffinized with xylene, washed with ethanol, and rehydrated. After hematoxylin and eosin staining, slides were imaged using a BZ-X810 microscope (Keyence) at 10× or 20× and stitched to obtain whole and detailed lung regions.

### Quantification of fungal burden by qPCR

Total genomic DNA was isolated from murine lung tissue using the DNeasy Tissue Kit (Qiagen). The fungal burden was determined by qPCR using primers and probes for the *Aspergillus* 18S rRNA gene (45). Cq values were compared to a six-point standard curve (lung tissue homogenates spiked with 10^2^–10^7^ conidia) to determine the number of conidial equivalents per lung.

### Analysis of bronchoalveolar lavage fluid

Sampling of BALF was performed by intratracheal instillation and collection of 1 mL of sterile PBS twice using a 20G cannula. Cytocentrifugation of 200 µL of lavage fluid was performed at 300 × *g* for 5 minutes using a Cytospin 4 centrifuge (Thermo Fisher Scientific). BALF samples were imaged in an automated BZX-810 microscope (Keyence) at 40×. Cell counting was performed in 2–3 fields of view from independent mice from each experimental group. Cells in contact with the image edge, blurred cells with poorly defined borders, and large or dense clumps of cells were excluded. Leukocyte cells were differentially counted as macrophages, monocytes, lymphocytes, and granulocytes (neutrophils or eosinophils). Total red blood cell counts included both erythrocytes and echinocytes (Burr cells).

### Transcriptional analyses

Lungs were weighed and flash-frozen in 1 mL RNAprotect Tissue Reagent (Qiagen). For RNA isolation, 1 mL RLT Buffer (Qiagen) was added to the thawed lungs, and tissue was homogenized using a Mini Bead-Beater (Biospec Products) and 15 acid-washed 3-mm glass beads (Sigma-Aldrich). Total RNA was isolated from a volume of lung homogenate equivalent to 30 mg of tissue using the RNeasy Tissue Kit and RNase-Free DNase Set (both Qiagen) according to the manufacturer’s instructions. The yield and purity of RNA were determined using a NanoDrop One^C^ spectrophotometer (Thermo Fisher Scientific).

The expression of 785 immune-related genes was then assessed using the murine Host Response nCounter panel (Nanostring Technologies). Data were analyzed using the nSolver Analysis Software, with background thresholding to the median of negative controls and normalization to the panel’s 12 housekeeping genes (geometric mean). Cell profiling of known ratios of immune cell type markers was used to characterize cell abundance scores based on the NanoString Cell Type Profiling Module (46). Gene identifiers and pairwise mean inter-group expression ratios were imported into Ingenuity Pathway Analysis (Qiagen). Core analysis was performed to study canonical pathway enrichment, considering genes with mean inter-group expression fold changes of >|1.25| for infection-induced differences and >|1.5| for therapeutic effects. Differential enrichment of canonical pathways was considered significant at an absolute *z*-score ≥ 1 and a Benjamini-Hochberg adjusted *P* value < 0.05.

To measure transcript levels of individual genes of interest (24, 30, 47, 48), we performed quantitative reverse transcription-PCR. About ~500 ng of total RNA was reverse transcribed using an iScript cDNA synthesis kit. RT-PCR was performed with SYBR Green PCR master mix using a CFX Connect Real-Time PCR Detection System (Bio-Rad). Relative mRNA expression was calculated by using a ∆∆Ct method relative to a housekeeping gene (GAPDH) and normalized to a control group (49). The following genes of interest were included: CCL2, DUOX2, IFN-γ, IL-6, IL-17A, SAA3, and TNF-α.

### Flow cytometry

Single-cell lung suspensions were prepared from disaggregated lungs, as previously reported (18). Briefly, murine lungs were harvested and stored in sterile PBS on ice for immediate processing. Lungs were cut into ≤1 mm^3^ pieces using razor blades and digested with collagenase/DNase I at 5 mg/mL for 45 minutes at 37°C. The resulting single-cell suspensions were passed through a 70 µm filter and washed with PBS. After red blood cell lysis, single-cell suspensions were washed with PBS + 1% fetal bovine serum and stained for specific cell markers. Antibody panels are summarized in Table S2.

For conventional flow cytometry, cells were fixed and subsequently acquired on a BD LSRFortessa X-20 (BD Biosciences) flow cytometer. Approximately 500,000 viable singlets were acquired per specimen and panel. Downstream analysis was performed using FlowJo version 10.8.1 (FlowJo, LLC). The gating strategy is summarized in Fig. S5.

For imaging flow cytometry, surface and intracellular staining were performed as previously reported (49). After antibody staining, cells were pelleted and resuspended in 50 µL of sterile PBS + 0.5 µg/mL nuclear DAPI stain. Thereafter, samples were measured on an ImageStreamX MII device using the INSPIRE software (Amnis Corporation) at 20× magnification with standard laser configurations. Channels 1 and 9 were used for brightfield, 6 for side scatter, and the others (2–5, 7, 8, 10–12) for fluorescent image capture. Single-color controls were captured for each fluorescent signal, and compensation was performed using the compensation wizard and IDEAS 6.1 software (Amnis Corporation). The gating strategy is summarized in Fig. S6.

### Cytokine measurements (Luminex assay)

Lung tissue was homogenized by bead beating in 1 mL PBS, as described above. A 13-plex magnetic murine cytokine and chemokine panel (LXSAMSM-13) was performed according to the manufacturer’s instructions (R&D Systems) and analyzed using a Luminex 200 device (Luminex Corporation). The following analytes were included: CCL2, CCL3, CCL4, CXCL2, GM-CSF, IFN-γ, IL-2, IL-4, IL-6, IL-12 p70, IL-17A, IL-33, and TNF-α.

### Statistical analyses

Survival curves and event-free survival were compared using the Mantel-Cox log-rank test. Results of immunoassays were compared using Student’s two-sided *t*-test or Mann-Whitney *U* test for two-group comparisons and one-way ANOVA with Tukey’s post-test or Kruskal-Wallis test with Dunn’s post-test for four-group comparisons. Significance tests are specified in the figure legends. Significant findings are denoted by asterisks: *P < 0.05, **P < 0.01, and ***P < 0.001. Numeric values are provided for *P*-values between 0.05 and 0.1. Data were analyzed and visualized using Excel 365 (Microsoft Corporation) and Prism version 9 (GraphPad Software).

## SUPPLEMENTAL MATERIAL

Supplemental material mbio.04061-24-s0001.docx Supplemental tables and figures.

ASM does not own the copyrights to Supplemental Material that may be linked to, or accessed through, an article. The authors have granted ASM a non-exclusive, world-wide license to publish the Supplemental Material files. Please contact the corresponding author directly for reuse.
