# Supplementary material for: Immunotherapy with nebulized pattern recognition receptor agonists restores severe immune paralysis and improves outcomes in mice with influenza-associated pulmonary aspergillosis
Source: mBio. 2025 Apr 8;16(5):e04061-24. doi: 10.1128/mbio.04061-24 (PMC12077147; doi:10.1128/mbio.04061-24)
Supplement: Supplemental material — Supplemental tables and figures. [file mbio.04061-24-s0001.docx]

**Immunotherapy with nebulized pattern recognition receptor agonists restores severe immune paralysis and improves outcomes in mice with influenza-associated pulmonary aspergillosis**

Supplementary Materials

**Table S1. Cytokine concentrations in lung tissue of mice with IAPA according to the treatment arm.**

All concentrations, including the LOD, are provided as pg per g of lung tissue. N = 3 mice per treatment arm.

| **Treatment ►** | | **PBS / PBS** | **Pam2ODN / PBS** | **Pam2ODN / Pam2ODN** | | |
| --- | --- | --- | --- | --- | --- | --- |
| **Analyte** | **LOD ^a^** | **Mean** | **Mean** | **Mean** | **Ratio vs. PBS / PBS** | **Ratio vs. Pam2ODN / PBS** |
| CCL2 | 389.2 | 1962.5 | # (2019.7) | 2488.0 | 1.27 | # (1.23) |
| CCL3 | 1.0 | 123.4 | 138.0 | 811.0 | 6.57 | 5.88 |
| CCL4 | 181.5 | Below the LOD in most mice or in at least one mouse per group. | | | | |
| CXCL2 | 4.9 | 207.5 | 148.0 | 776.7 | 3.74 | 5.25 |
| GM-CSF | 10.3 | 29.9 | 25.1 | 60.1 | 2.01 | 2.39 |
| IFN-γ | 17.4 | Below the LOD in most mice or in at least one mouse per group. | | | | |
| IL-2 | 10.0 | 15.2 | 18.8 | 20.7 | 1.36 | 1.11 |
| IL-4 | 59.8 | 248.6 | 371.0 | 179.5 | 0.72 | 0.48 |
| IL-6 | 28.8 | Below the LOD in most mice or in at least one mouse per group. | | | | |
| IL-12 p70 | 46.8 | Below the LOD in most mice or in at least one mouse per group. | | | | |
| IL-17A | 45.2 | Below the LOD in most mice or in at least one mouse per group. | | | | |
| IL-33 | 103.9 | 30649.7 | 27699.2 | 38432.2 | 1.25 | 1.39 |
| TNF-α | 2.1 | Below the LOD in most mice or in at least one mouse per group. | | | | |

^a^ 10% of the lowest standard at 100 mg lung weight.

Yellow highlights denote cytokines that showed significant differences in 3-group comparisons (Kruskal-Wallis test) and were therefore included in **Fig. 3B**.

#: Mean and ratio not reliably determinable due to one measurement below the LOD. Numbers in parentheses indicate the mean when excluding the single low outlier.

Abbreviations: C(X)CL = C-(X-)C motif chemokine ligand, GM-CSF = Granulocyte-macrophage colony-stimulating factor, IAPA = influenza-associated pulmonary aspergillosis, IFN = interferon, IL = interleukin, LOD = limit of detection, PBS = phosphate-buffered saline, Pam2ODN = Pam-2 CSK4 + CpG oligodeoxynucleotides M362, TNF-α = tumor necrosis factor alpha.

**Table S2. Flow cytometry antibodies and reagents.**

| **Materials / Resources** | **Reference/Identifier** | **Source** |
| --- | --- | --- |
| ***Conventional Flow Cytometry: Myeloid Antibody Panel*** | | |
| CD45 (30-F11) – redFluor 700 | Cat # 80-0451-U100 | Tonbo Biosciences |
| CD11b (M1/70) – PerCP-Cy5.5 | Cat # 45-0112-82 | Invitrogen |
| CD11c (N418) – Brilliant Violet 421 | Cat # 117330 | BioLegend |
| MHC II (M5/114.15.2) – PE-Cy 7 | Cat # 107630 | BioLegend |
| F4/80 Antigen (BM8.1) – APC-Cy 7 | Cat # 25-4801-U100 | Tonbo Biosciences |
| Ly-6 C (HK1.4) – APC | Cat # 128016 | BioLegend |
| Ly-6 G (1A8) – Alexa Fluor 488 | Cat # 127626 | BioLegend |
| CD24 (M1/69) – PE | Cat # 50-0242-U100 | Tonbo Biosciences |
| CD64 (X54-5/7.1) – Brilliant Violet 711 | Cat # 139311 | BioLegend |
| Siglec-F (E50-2440) – PE-CF594 | Cat # 562757 | BD Biosciences |
| Ghost Dye Violet 510 | Cat # 13-0870-T100 | Tonbo Biosciences |
| ***Conventional Flow Cytometry: T Cells Antibody Panel*** | | |
| CD3e (145-2C11) – PerCP-Cy 5.5 | Cat # 65-0031-U100 | Tonbo Biosciences |
| UCD4 (RM4-5) – PE-Cy 7 | Cat # 60-0042-U100 | Tonbo Biosciences |
| CD8a (53.6.7) – APC-Cy 7 | Cat # 25-0081-U100 | Tonbo Biosciences |
| CD44 (IM7) – Brilliant Violet 711 | Cat # 103057 | BioLegend |
| CD62L (MEL-14) – Brilliant Violet 421 | Cat # 104435 | BioLegend |
| Ghost Dye Violet 510 | Cat # 13-0870-T100 | Tonbo Biosciences |
| CD45 (30-F11) – redFluor 700 | Cat # 80-0451-U100 | Tonbo Biosciences |
| ***Imaging Flow Cytometry: Structural Cells Antibody Panel*** | | |
| DAPI solution, 1mg/mL | Cat # 62248 | Thermo Fisher Scientific |
| CD45 (30-F11) – Brilliant Violet 605 | Cat # 103140 | BioLegend |
| CD324 (DECMA-1) – PE-Dazzle 594 | Cat # 147316 | BioLegend |
| Gp38 (8.1.1) – PerCP-Cy 5.5 | Cat # 127422 | BioLegend |
| CD31 (MEC13.3) – APC-Cy 7 | Cat # 102534 | BioLegend |
| NF-kB p65 (F-6) – Alexa Fluor 488 | Cat # sc-8008 AF488 | Santa Cruz  Biotechnology |
| c-Jun (G-4) – Alexa Fluor 647 | Cat # sc-74543 AF647 | Santa Cruz  Biotechnology |

Abbreviations: CD = Cluster of Differentiation; PerCP = Peridinin-Chlorophyll-Protein; MHC = Major Histocompatibility Complex; Ly-6 = Lymphocyte Antigen 6 Complex; Siglec = Sialic Acid Binding Ig-like Lectin; PE = Phycoerythrin; Cy = Cyanine; CF = Cyanine-based Fluorescent; APC = Allophycocyanin.

**Figure S1 (caption on following page)**

**Figure S1: Absolute normalized transcript counts of representative epithelial and immune-related genes in lung tissue homogenates of mice with IAPA compared to those with IAV infection only.**

Data has been obtained using the nCounter Host Response panel and is based on the same specimens (n = 3 per group) used for **Fig. 1B-D** following the experimental design shown in **Fig. 1A**.


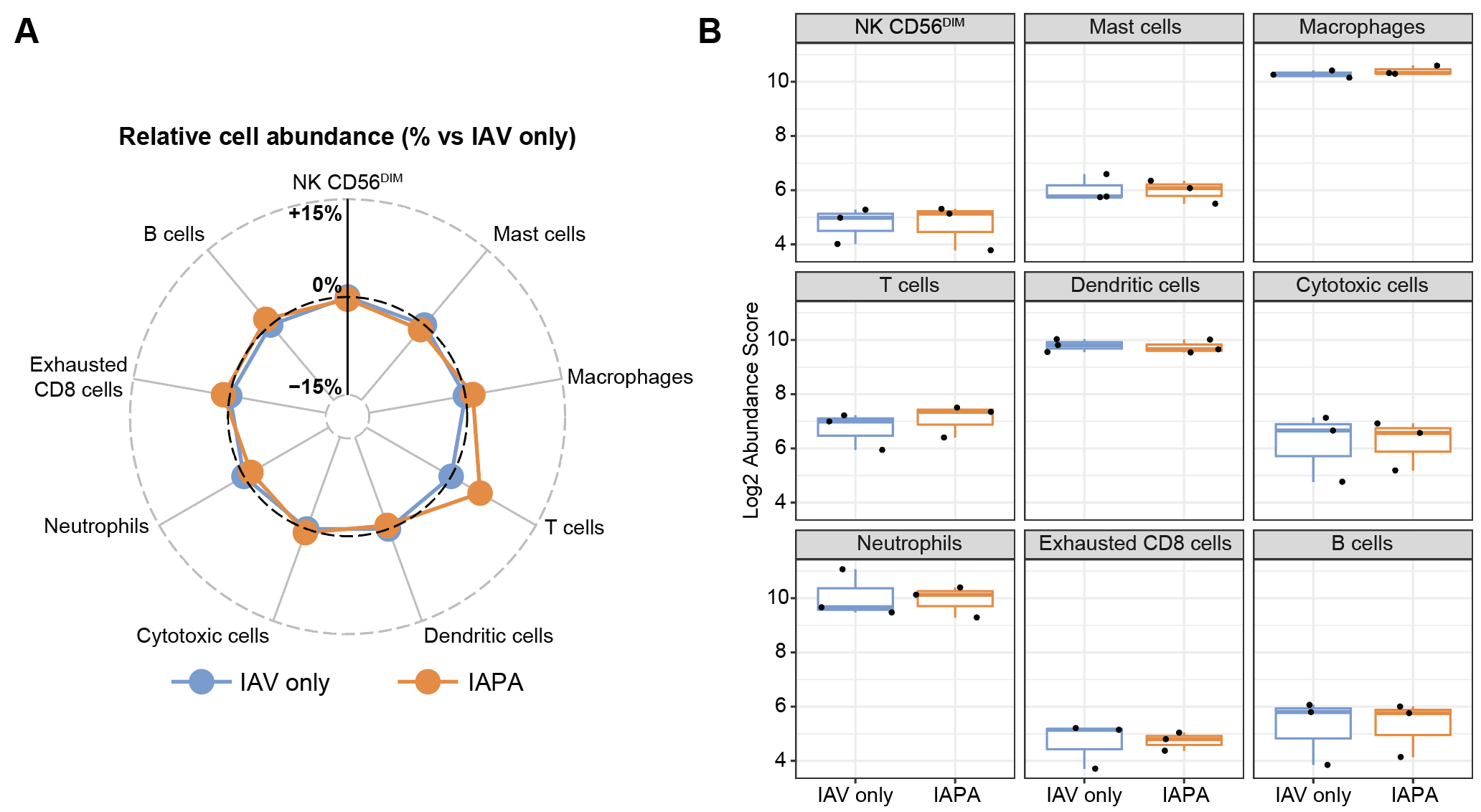


**Figure S2: Leukocyte abundance in lungs of mice with IAPA or IAV infection only. (A)** Radar plot of relative immune cell abundance (percentual change) in lung tissue of mice with IAPA versus those with IAV infection only. **(B)** Boxplot of log_2_ abundance scores per cell type and group. Abundance scores were estimated by nCounter-based cell type profiling analysis (ROSALIND Bio). Data is based on the same specimens (n = 3 per group) used for **Fig. 1B-D** following the experimental design shown in **Fig. 1A**. Abbreviations: CD = cluster of differentiation, IAV = influenza A virus, IAPA = influenza-associated pulmonary aspergillosis, NK = natural killer cells.

**Figure S3 (caption on following page)**

**Figure S3: Dual-dose Pam2ODN immunotherapy induces a transcriptional framework promoting recruitment of mononuclear effector cells to the lungs of mice with IAPA.**

Networks of transcriptional changes to the pulmonary immune environment in mice receiving dual-dose Pam2ODN therapy compared to those receiving either single-dose Pam2ODN (**A**) or mock therapy (**B**), as predicted by Ingenuity Pathway Analysis. N = 3 mice per treatment arm.

Abbreviations: C(X)CL = C-(X-)C motif chemokine ligand, IAPA = influenza-associated pulmonary aspergillosis, IFN = interferon, IKBKB = inhibitor of nuclear factor kappa B kinase subunit beta, IL = interleukin, MYD88 = myeloid differentiation primary response 88, NK = natural killer cells, PBS = phosphate-buffered saline, Pam2ODN = Pam-2 CSK4 + CpG oligodeoxynucleotides M362, RELA = v-rel avian reticuloendotheliosis viral oncogene homolog A, TICAM1 = TIR domain containing adaptor molecule 1, TLR = Toll-like receptor.

**Figure S4 (caption on following page)**

**Figure S4: Pam2ODN immunotherapy attenuates early interferon gamma expression and induces epithelial resistance markers.**

(**A**) Timeline of experimental interventions. (**B**) Expression of selected epithelial and immune-related genes on day 10 in mice with IAPA that received Pam2ODN versus those that received mock therapy with PBS, as determined by qPCR. N = 5 mice per treatment arm. Unpaired t-test. Abbreviations: AF = *Aspergillus fumigatus*, CA = cortisone acetate, IAPA = influenza-associated pulmonary aspergillosis, IAV = influenza A virus, PBS = phosphate-buffered saline, Pam2ODN = Pam-2 CSK4 + CpG oligodeoxynucleotides M362, qPCR = quantitative polymerase chain reaction.

**Figure S5 (caption on following page)**

**Figure S5: Gating strategy for conventional flow cytometry.**

Viable leukocytes (CD45^+^ viable singlets) were identified by eliminating debris (low FSC-A), gating on the FSC-H/FSC-A diagonal, exclusion of dead cells (ghost dye >50k fluorescence units), and selection of the CD45^+^ population. For myeloid cell gating, neutrophils were first identified by Ly6G positivity. Among the remaining cells (R4), CD11b^low^ CD11c^low^ cells were excluded (R6). The remaining cells (R5) were sub-divided based on granularity (SSC-A) and MHC-II expression. Among SSC-A^low^ MHC-II^low^ cells (R7), monocytes (CD11b^+^CD64^+^) and NK cells (CD11b^+^CD64^-^) were identified. Among SSC-A^high^ and/or MHC-II^+^ cells (R8), macrophages (CD24^low^) were identified and sub-divided into alveolar macrophages (AMO) and interstitial macrophages (IMO) based on CD11b expression. CD24^bright^ cells (R9) were sub-divided into eosinophils (CD11b^+^MHC-II^-^), CD11b^-^ dendritic cells (CD11b^-^MHC-II^+^), and CD11b^+^ dendritic cells (CD11b^+^MHC-II^+^). Cells in R6 and unclassified cells in R7 were considered lymphocytes. After subtraction of the percentage of CD3^+^ T cells among viable CD45^+^ viable singlets (lymphocyte panel), the remainder was reported as “B cells and other lymphocytes”. For T-cell sub-differentiation (not reported in the manuscript), T-helper (Th) cells (CD4^+^) and cytotoxic lymphocytes (CD8^+^) were identified. Th cells were further differentiated into naïve/stem cell memory Th cells (CD44^-^ CD62L^+^), central memory Th cells (CD44^+^ CD62L^+^), effector memory and effector Th cells (CD44^+^ CD62L^-^), and terminally differentiated Th cells (CD44^-^ CD62L^-^). Gating was performed with FlowJo v10.8.1 using 5% contour plots and histogram plots.

**Figure S6: Gating strategy for imaging flow cytometry.**

Single-nucleated cellular singlets for all cell types were identified from single-cell lung suspensions using a pan-cellular gating approach by eliminating debris and beads (G1, excluding low area and variable SSC), selection of cell singlets with high whole-cell (Ch09) aspect ratio (G2), selection of in-focus cell populations (G3; high gradient RMS for both whole cells [Ch09] and nuclei [Ch07]), selection of nucleated (mid-to-high DAPI) cells (G4) to exclude remaining non-nucleated cells (i.e., erythrocytes), and selection of single nucleated (non-dividing) cells (G5) with high nuclear (Ch07) aspect ratio. Cells were then sub-divided into 4 distinct cell types using a cell-specific gating approach: First, leukocytes were identified by CD45 positivity (G6). Among the remaining cells (G7), epithelial cells (G8) were identified by CD324 (E-Cadherin) positivity. Among the remaining cells (G9), fibroblasts (G10) were identified by Gp38 (E-Cadherin) positivity and mutual exclusion from endothelial cells (G11) identified by CD31 positivity.
